# Flexible 3D Force Sensor Based on Polymer Nanocomposite for Soft Robotics and Medical Applications

**DOI:** 10.3390/s24061859

**Published:** 2024-03-14

**Authors:** Ahmed Alotaibi

**Affiliations:** Department of Mechanical Engineering, College of Engineering, Taif University, Taif 21944, Saudi Arabia; am.alotaibi@tu.edu.sa

**Keywords:** flexible, 3D force sensor, tactile, polymer nanocomposite, strain, measurements

## Abstract

The three-dimensional (3D) force sensor has become essential in industrial and medical applications. The existing conventional 3D force sensors quantify the three-direction force components at a point of interest or extended contact area. However, they are typically made of rigid, complex structures and expensive materials, making them hard to implement in different soft or fixable industrial and medical applications. In this work, a new flexible 3D force sensor based on polymer nanocomposite (PNC) sensing elements was proposed and tested for its sensitivity to forces in the 3D space. Multi-walled carbon nanotube/polyvinylidene fluoride (MWCNT/PVDF) sensing element films were fabricated using the spray coating technique. The MWCNTs play an essential role in strain sensitivity in the sensing elements. They have been utilized for internal strain measurements of the fixable 3D force sensor’s structure in response to 3D forces. The MWCNT/PVDF was selected for its high sensitivity and capability to measure high and low-frequency forces. Four sensing elements were distributed into a cross-beam structure configuration, the most typically used solid 3D force sensor. Then, the sensing elements were inserted between two silicone rubber layers to enhance the sensor’s flexibility. The developed sensor was tested under different static and dynamic loading scenarios and exhibited excellent sensitivity and ability to distinguish between tension and compression force directions. The proposed sensor can be implemented in vast applications, including soft robotics and prostheses’ internal forces of patients with limb amputations.

## 1. Introduction

Strain sensors have recently attracted tremendous attention due to their ability to measure a wide range of physical quantities such as stress, strain, pressure, torque, and vibration. That necessitated an extensive investigation and comparison of the different strain sensing technologies to elucidate their advantages and disadvantages. Some of these existing strain sensing technologies include resistive [1], capacitive [2], optical [3], and piezoelectric transducers [4]. The need for flexible strain sensors is growing rapidly, especially for skin-mountable wearable electronics and structural health monitoring applications [5,6]. Flexible force sensors measure one-direction (1D) contact pressure force for robotic and medical applications. These sensors can assist robots in determining their grasp strength, avoiding excessive force, and recognizing the grasped object materials. In addition, they are utilized to miniaturize different intelligent medical systems, which gives them great potential for health monitoring wearable devices [7]. However, 1D force sensors limit the available 3D measurement information during operation in real-world applications where multidirectional forces play a critical role [8].

The three-dimensional (3D) force and pressure mapping sensors have become essential in many industrial and medical applications. The three force components in the 3D space and force/pressure distribution need to be acquired. In industry, 3D force measurement helps robot arms operate under the allowable and safe force/torque range. In addition, it assists computer numerical control (CNC) machines in averting tool deflection and machine failure during the machining process. In the medical field, robot-assisted surgery uses 3D force/torque sensors to monitor the surgical operation remotely and avoid excessive forces. Therefore, there is a need to develop a flexible sensor for delicate operations and limited placements in the industrial and medical sectors.

Several attempts have been made to develop flexible tactile sensors for 3D force measurement. These sensors utilized different strain measurement technologies, including capacitive, piezoresistive, and piezoelectric characteristics. A considerable amount of literature has been focused on capacitive-based flexible tactile 3D force sensors. Viry et al. introduced a flexible tactile three-axial force sensor [9]. It consists of three dielectric layers: the fluorosilicone, air gap, and shed created by the woven fabric electrodes. The electrodes and dielectric layers were encapsulated between two polydimethylsiloxane (PDMS) layers to enhance the sensor’s flexibility. The sensor shows good sensitivity; however, the maximum force detection is limited to 400 kPa (ca. 27 N), which is imposed by the fluorosilicone layer’s maximum operating stress [10,11]. In addition, a maximum applied force of 12 N (190 KPa) was tested only in this work [9].

Various multi-layered tactile sensor arrays with four capacitors sandwiched between two polydimethylsiloxane (PDMS) flexible layers were introduced in the literature [12,13,14,15,16,17,18]. Lee et al. developed a tactile sensor array with the capability of measuring both normal and shear forces [12,13,14]. The capacitors’ electrodes were separated by air gaps, where the applied forces influenced the distance between electrodes. An external upper bump enhanced the sensor sensitivity to literal forces. This was tested for a small force range of 20 mN with an average sensitivity of 1.23%/mN along the 3D axes [13]. Similarly, Cheng et al. used a comparable structure configuration and fabricated the proposed sensor using micromachining techniques and flexible printed circuit board (FPCB) technologies [15,16]. The maximum recorded sensitivity was 1.67%/mN under a limited force range of 26 mN [15]. Polymer nanocomposites have been utilized in a capacitive-based flexible tactile sensor for 3D force measurements [18]. The sensor consisted of four upper electrode layers and a common lower electrode, both made of carbon nanotube/polydimethylsiloxane (CNT/PDMS) composite layer. The sensor electrodes were separated by carbon black/polydimethylsiloxane (PDMS) composite layer. The sensor demonstrated resolutions 0.1 N and 0.2 N under 50 N of normal force and 10 N of tangential direction. The capacitive-based sensor is suspectable to noise, especially when it comes in contact with the human body [18].

Several piezoresistive-based flexible 3D force sensors were introduced in the literature. A pressure-based flexible 3D force sensor was fabricated, and Velostat films were utilized as their sandwiched sensing elements [19]. The polydimethylsiloxane (PDMS) bump was implanted on the sensor structure to protect the sensor’s structure and convey force measurements. The proposed sensor measured the maximum force of 15 N and 5.5 N in the normal and shear force directions, respectively. A good cyclic performance was achieved at a narrow operating frequency of 0.5–2 Hz under a maximum force of 8 N. That limited the sensor’s application to low frequency and force application. Similar to the previous study, a compression-based flexible 3D force sensor was constructed utilizing the carbon black on polyimide material (PI) encapsulated in PDMS [20]. The sensor performed comparably to the previous work with relatively higher force ranges [19]. Similarly, NiCr sensing element-based 3D force-sensing, which utilized the piezoresistive characteristic of NiCr and their resistance changes under an applied force, was introduced [21]. Nevertheless, the sensor was tested at narrow force ranges of 0–300 mN and 300–500 mN in the shear and normal directions, respectively. In addition, the sensor indicated distinct sensitivity behaviors in the narrow normal direction force range. Hwang et al. utilized four strain gauges between polyimide and polydimethylsiloxane substrate layers [22]. The sensor incorporated the bump structure for load distribution. The four sensing elements measured the normal and shear forces through the deformation of the polymer substrate under loading. The developed sensor could measure forces up to 4 N in shear and normal loading and saturated under high forces.

A 3D force, based on micromachined single crystal silicon, was introduced and analyzed for its capability to quantify forces [23]. The sensor used a cross-structure design and flexible hinges for its elastic structure. In addition, the p-doped resistors were utilized as sensing elements and attached to the membrane hinges. The sensor was only tested under normal force, and the maximum force range was restricted to 0.5 N. That resulted from the maximum allowable displacement of the structure under normal force. In another study, the polyvinylidene fluoride (PVDF) piezoelectric characteristic was used to develop a flexible tactile sensor for 3D force measurements [24]. The sensor implanted the conventional bump structure with six aluminum (Al) electrodes serving as the upper electrodes and one for the lower common electrode. The PVDF layer’s generated output charges under the loading on the tactile sensor were captured using the implanted electrodes. The sensor was tested under dynamic loading with forces ranging from 0 to 0.5 N in the X and Y axes and 0 to 1.5 N in the Z axis. The sensor demonstrated good sensitivity with 14.9 pC/N in the x and y axes and 6.62 pC/N in the normal diction.

For people with lower limb amputation, the pressure distribution between the socket and residual limb is essential in patient discomfort and prosthetic limb performance. Pressure and stress inside the socket-residual limb could damage the skin and affect the internal tissues [25]. Several parameters influence the force distribution on the patient’s limb, such as the suspension system, socket fit, and alignment [26,27,28,29]. Several researchers have utilized different pressure/stress measurement strategies and techniques to quantify the pressure distribution.

A commercially available TEKSCAN 9810 pressure transducer was tested for stump/socket interface pressure mapping [30]. The sensor was based on Force Sensing Resistor (FSR) technology and contained 96 FSR cells. The sensor failed to match measured pressure with a 10% error and suffered sensitivity to loading rates and hysteresis errors. The internal curvature probably introduced the inaccuracy of measurements inside the prosthetic socket and the inability to adapt to that curvature shape.

A modular prosthesis pyramid connector was introduced to evaluate the socket reaction moments at different alignments [26,27,28,29]. The system is connected below the socket and does not quantify forces or pressure inside the socket. Most available pressure mapping measurement systems are complex, expensive, and complicated lab setups [31]. There is a need to produce a compact real-time 3D force/pressure measurement system to assess the connection between the residual limb and liner. The new measurement system should be flexible and can adapt to different limb shapes. In addition, the sensor should be compact to be embedded in the socket or liner. That would minimize the patient’s discomfort, minimize clinical visits to adjust the prosthetic limbs, and improve the prosthetic limb’s performance and fitting.

The MWCNT/PVDF nanocomposite films are unique sensing elements that can capture both dynamic and static strain due to the presence of both piezoelectric and piezoresistive properties, respectively [32]. The electrical conductivity of PNCs can be enhanced by a lower proportion of CNTs [33]. Furthermore, PNC-CNTs attained higher sensitivity than typical strain gauges [34]. The piezoresistive characteristics of such composites are affected by tunneling, crossing, and CNT resistance [35]. The tunneling and CNT resistance are the most and least influencing factors in the film’s overall conductivity, respectively [36,37]. On the other hand, PVDF has been utilized in different biomedical applications due to its unique piezoelectric characteristics, chemical resistance, thermal stability, and mechanical properties [38]. The MWCNT/PVDF film’s percolation threshold, where electrical conductivity significantly increased, was around 1.5 wt.% [39]. In addition, the maximum conductivity was achieved in the concentration of 4–5 wt.%. The highest gauge factor (GF) of similar composite SWANT/MWCNT films was 6.2, and the percolation threshold was 2 wt.% [40]. Meanwhile, the gauge factor for metallic folies typically ranges between 2 and 5 [41]. That offers an advantage over conventional strain sensors due to the adjustability of the sensor’s sensitivity and measurement ranges using the CNT’s contents percentage weight [42]. In addition, these PNC-CNTs can be deposited on a flexible substrate, which leads to a mechanical flexible strain sensor. In contrast, conventional strain sensors have various limitations, including limited measurement range, lower sensitivity, rigid structure, and sensitivity to fatigue and environmental conditions [42].

**Figure 1:**
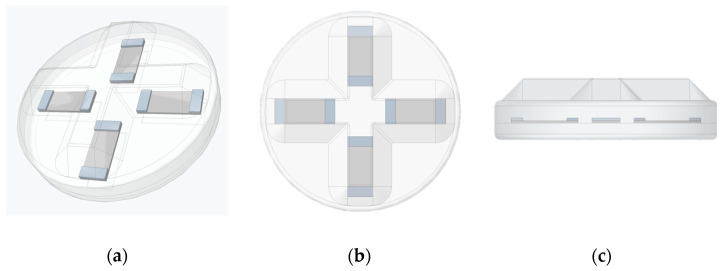
The proposed flexible 3D force sensor’s CAD design (**a**), top view (**b**), and side view (**c**).

In this study, a novel flexible 3D force sensor-based polymer nanocomposite sensing is introduced in Figure 1a. This sensor can be implemented in industrial and medical applications, including soft robotic and prosthetic limbs. It consists of four nanocomposite sensing elements arranged in a cross-beam structure and sandwiched between two layers of silicone rubber, as shown in Figure 1b,c. The sensing elements were made of multi-walled carbon nanotubes and polyvinylidene fluoride (MWCNT/PVDF) and fabricated using the spray coating technique. The MWCNT/PVDF sensing elements were selected for their high sensitivity. In addition, they can capture both static and dynamic strain measurements using piezoresistive and piezoelectric characteristics [32].

## 2. Sensor Design and Fabrication

The development of the flexible 3D force sensor involved two consecutive fabrication processes. They start with the fabrication of MWCNT/PVDF sensing elements, followed by the flexible 3D force sensor construction utilizing the fabricated sensing elements. The fabrication processes of the proposed flexible 3D force are presented in the following subsections.

### 2.1. Sensing Elements Fabrication

**Figure 2:**
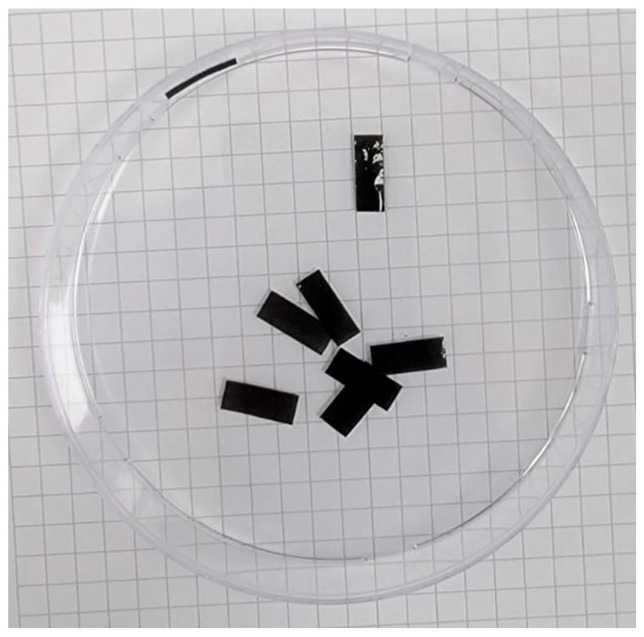
The fabricated MWCNT/PVDF sensing elements.

The MWCNT/PVDF nanocomposite was selected as the sensing elements of the proposed flexible 3D force sensor. Solution mixing and spray coating techniques were used to fabricate the strain sensing elements, as described in the literature [32,43]. The PNC-CNT films with lower CNT concentrations result in high electrical resistance films, which lead to more complex signal conditioning than metallic strain gauges [42]. As a result, 4 wt. % of MWCNTs (20 mg) were chosen and mixed with 50 mL of N-N dimethylformamide (DMF) to ensure the CNTs’ dispersion and avoid CNTs aggregation. The mixture was placed in a sonication path for 30 min at room temperature. At the same time, 0.5 g of PIVDF was dissolved in 5 mL of DMF and mixed in a magnetic stirrer for three hours at a temperature of 80°. Then, the two mixtures were blended at room temperature until a homogenous solution was achieved. The resultant solution was sprayed on glass substrates using an airbrush at room temperature. The MWCNT/PVDF sensing elements were peeled off the substrates by immersion in the sonication bath. The average thickness of the resulting films was measured at approximately 26 μm. Finally, the resulting films were placed on a hot plate at 80° to enhance the solvent evaporation. Four sensing $12\times 5\;{\mathrm{mm}}^{2}$ elements were cut to be implemented in the developed flexible 3D force sensor as their sensing elements, as shown in Figure 2.

### 2.2. Flexible 3D Force Sensor Fabrication and Operation Principle

**Figure 3:**
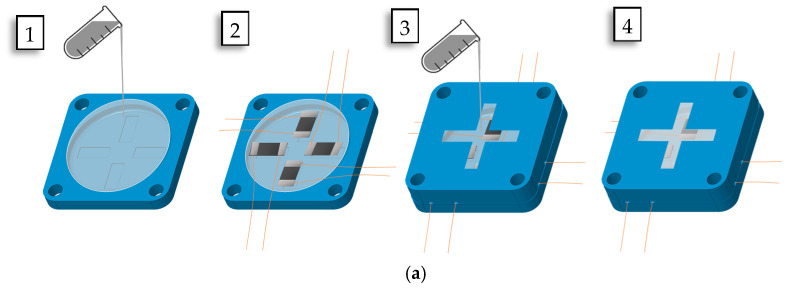

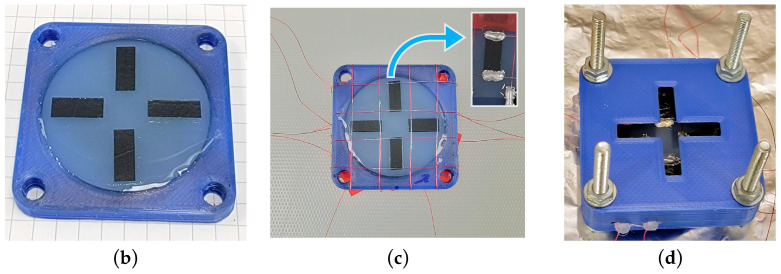
The 3D flexible sensor fabrication mold process. (**a**) Fabrication mold process schematic. (**b**) Sensor lower part with sensing elements attached. (**c**) Wires were attached to the sensor’s lower parts and attached using Silver Epoxy electrodes. (**d**) The upper model was assembled and prepared for pouring the rubber mixture.

The 3D force sensor is usually built based on design elements. These elements include structure configuration and sensing elements placements. Both factors play an essential role in the performance of 3D force sensors. The cross-structure configuration, with sensing elements distributed on their four structural beams, is widely implemented in different industrial 3D force sensors [44]. In this study, a flexible 3D force sensor was proposed utilizing the cross-structure configuration’s sensor placements and fixable host matrix material, as shown in Figure 1. The flexible 3D force sensor was constructed using silicon rubber due to its flexibility and ease of fabrication. It is widely used in robotics and soft robotics applications. The sensor was designed with a diameter of 40 mm and a height of 11 mm. The lower and upper parts, excluding the bump structure, were 3 mm and 4 mm, respectively. The 4 mm bump structure was implemented to enhance the sensor’s sensitivity to literal forces, protect the sensor’s structure, and convey force measurements. The proposed 3D sensor was fabricated using the mold casting fabrication technique. The 3D printing technology was used to construct the required mold to achieve the desired sensor shape, as shown in Figure 1. The produced mold consisted of an upper and a lower mold. A summary of the fabrication process schematic is shown in Figure 3a.

**Figure 4:**
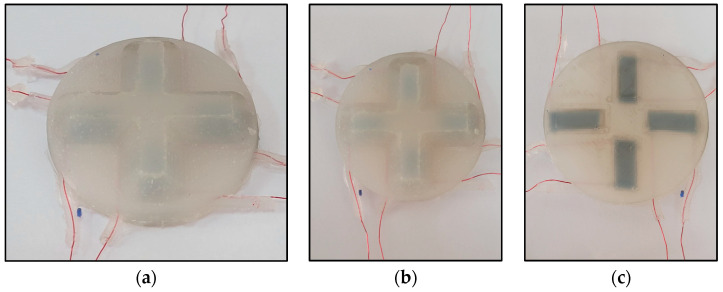
Fabricated 3D flexible sensor using mold casting fabrication technique. (**a**) Isometric view. (**b**) Top view. (**c**) Bottom view.

First, the silicone rubber mixture EcoflexTM 00-03, Smooth-On, Inc., Macungie, PA, USA, was prepared and poured into the lowered mold until it was filled, and it was left to cure for 24 h at room temperature. Then, the four sensing elements were allocated based on the cross-beam structure configuration 3D force sensor, as shown in Figure 3b. Enameled copper magnet wires with a diameter of 0.2 mm were fixed to the lower mold at the ends of each sensing element, as shown in Figure 3c. The wires’ coating was removed at contact points with each sensing element’s ends. The AA-DUCT 916 Flexible Silver Epoxy Adhesives (Atom Adhesives) electrodes were deposited at the ends of the sensing elements’ wires for measurement. The upper mold was set above the lower one and filled with the silicone rubber mixture again, as shown in Figure 3d. The copper wires were permitted to exit the upper mold through small side gaps. The molded flexible 3D force sensor was set at room temperature for 24 h until fully cured. Finally, the 3D force sensor was removed from the mold, and excessive materials were trimmed, as shown in Figure 4.

**Figure 5:**
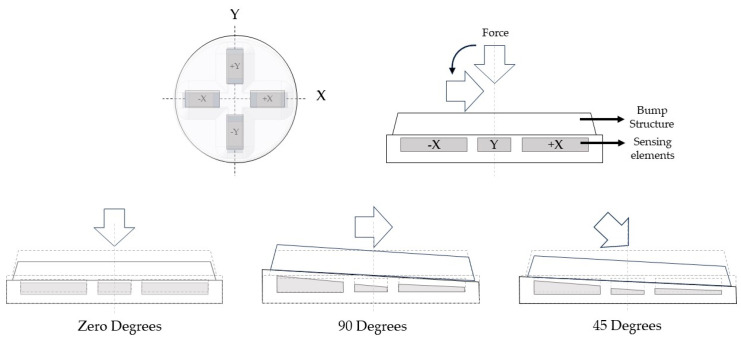
The 3D flexible sensor sensing elements’ alignment and operation principle.

The flexible 3D force sensor was tested at different force angles: 90°, 45°, and zero degrees. The sensor’s operation principle is illustrated in Figure 5. The bump structure is compressed at the normal force condition, and the four-sensing element experiences symmetrical deformation. When the bump structure experiences torque due to the applied shear force at an angle of 90° (shear force), the +X sensing element will be compressed while the −X sensing element is stretched. At the same time, the Y-sensing elements will experience the shear force effect in the +X axis direction. On the other hand, when an inclined force with an angle of 45° is applied to the flexible 3D force sensor, it will lead to the combined effect of both normal and shear force components. The compression and tension strain affect the CNTs’ positioning and resistivity. The MWCNTs get closer when the sensing element is under compression. As a result, more electrical connective channels are formed, and the sensing elements’ resistance decreases. In contrast, the resistivity of the sensing elements increases when they starch due to the MWCNTs moving further from each other.

## 3. Experimental Setup

**Figure 6:**
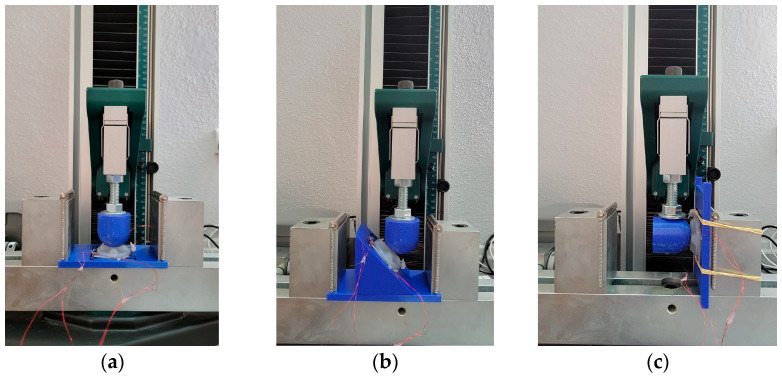
The fabricated 3D flexible sensor fabrication mold. (**a**) zero degrees (−Z direction). (**b**) 45 degrees (X direction). (**c**) 90 degrees (−X direction).

The flexible 3D force sensor’s performance and sensitivity were investigated under static and dynamic loading using a tensile/compression testing machine. The fabricated sensor was tested at different force angles: 90°, 45°, and zero degrees. These angles were measured between the loading direction and the sensor’s Z axis, as shown in Figure 6. Two PLA-loading spherical heads with a diameter of 40 mm were constructed using 3D printing technology and attached to the machine’s load cell using bolts and nuts. The first loading head was constructed to align with the machine load cell alignment for the zero and 45° loading tests, as shown in Figure 6a,b. On the other hand, the second loading head was attached perpendicular to the loading machine’s load cell to ensure 90° angle loading with the sensor’s Z axis, as shown in Figure 6c. Regarding the flexible 3D force sensor fixture, two 3D-printed fixture holders were designed to hold the sensor fixed during testing. The first flat holder attached to the machine clamping system at two different angles in the zero and 90° testing, as shown in Figure 6a,c. A 45° holder was combined with the former holed to maintain the sensor at the same angle during the sensor examination, as shown in Figure 6b.

**Figure 7:**
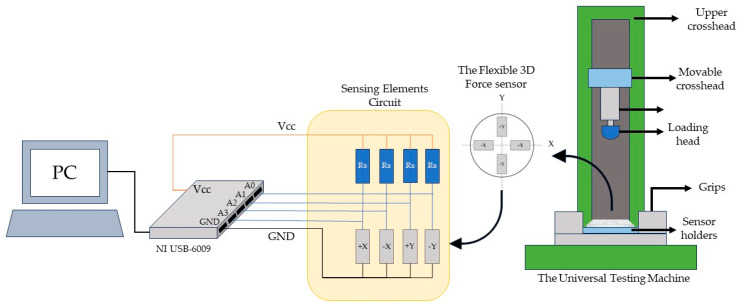
The experimental measurement setup and electrical circuit.

For the static testing, the applied force was increased gradually from 0 N to 25 N in the zero degree and 45° loading testing scenarios in the −Z axis and X axis directions, respectively. A maximum loading of 12 N was applied at the 90° testing setup in the −X axis direction. The relationship between the voltage readings of the four distributed sensing elements and the applied forces was retrieved and plotted. On the other hand, a dynamic loading with an acceleration of 0.167 mm/sec was applied to the flexible 3D force sensor. The sensor experienced loading forces in the same assigned direction for each setup, except for a different initial load that was applied to the sensor before testing to ensure complete contact between the loading head and the 3D flexible force sensor. A maximum loading of 22 N was applied in the −Z and X directions with angles of 0° and 45°, respectively. In the force shear loading test with an angle of 90° in the −X direction, the maximum applied force was 10 N. Four pairs of simple voltage dividers signal conditioning circuits were used to measure sensing elements’ resistance changes under applied external forces, as shown in Figure 7. The resistance Rs were selected to be equal to the paired sensing element to achieve high-sensitivity measurements. The voltage measurements of the four sensing elements circuit were acquired using a data acquisition (DAQ) system from National Instruments and presented using LabVIEW 2020 software. The sensing element voltage signals were sampled at a frequency of 1000 Hz, and a low pass filter with a cutoff frequency of 100 Hz was utilized to eliminate higher-frequency noise.

The voltage readings during the dynamic test were retrieved and plotted during the loading interval time. The sensors’ measurements were imported to MATLAB R2019b, and signal conditioning was performed using MATLAB R2019b’s signal analyzer tool. Further investigations using these measurements were conducted to assess the flexible 3D force sensor’s performance and sensitivity under static and dynamic loading scenarios in the following section.

## 4. Results and Discussion

The proposed flexible 3D force sensor was tested under static and dynamic loading conditions. These experiments were used to evaluate the effectiveness of this sensor in broad applications, including real-world industrial and medical applications. In addition, the sensor sensitivity was investigated. The following subsections discuss the experiment’s results in detail.

### 4.1. Static Loading Results

**Figure 8:**
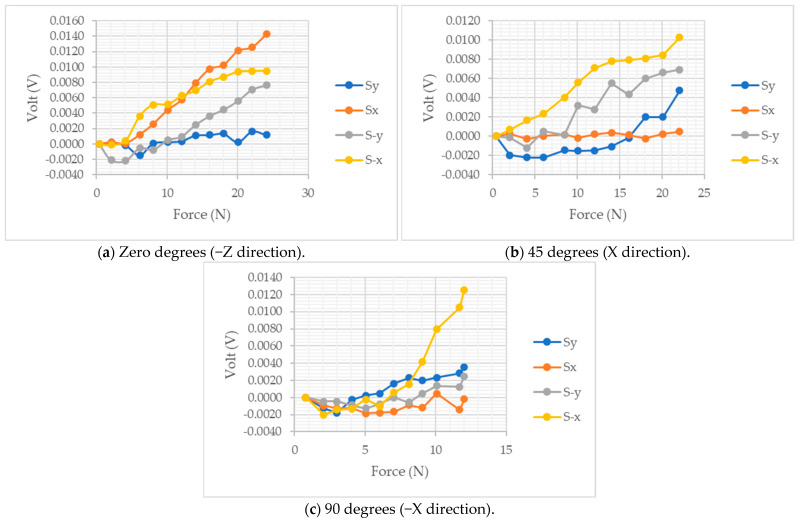
The flexible 3D force sensor static loading results.

The proposed sensor experienced static loading forces in three different directions at different engagement angles, and measurements were retrieved, as shown in Figure 8. The DC gains were removed from the voltage readings of the four sensing elements. Normal compression forces from 0 N to 22 N were applied to the sensor’s center, and all voltage readings were recorded, as shown in Figure 8a. The sensing elements at the +X axis recorded the highest sensitivity of 7 × 10^−4^ Volt/N. Meanwhile, the sensing elements at −X and −Y axes measured the force changes with a slightly lower sensitivity of 5 × 10^−4^ Volt/N and 4 × 10^−4^ Volt/N, respectively. On the other hand, the sensing element at the +Y axis was the least sensitive to the force changes with a sensitivity of 8 × 10^−5^ Volt/N; however, it still demonstrated an increase in pressure.

The fixable 3D force sensor was tested in the +X axis direction with a force at an angle of 45°, as shown in Figure 8b. The sensing elements at the −X and −Y axes attained similar sensitivity during normal compression testing. The −Y sensing element was expected to be less sensitive to force in the +X direction; however, the 3D force sensor might experience slight rotation toward the +Y direction, which could result in partial contact between the loading head and the −Y supporting structure. The sensitivity of the sensing elements at the +Y axis has increased to 2 × 10^−4^ Volt/N. However, the sensing element at the X axis was the least influenced, with a sensitivity of 1 × 10^−5^ Volt/N. That might be due to the influence of the bump structure or spherical head alignments. At the 90° shear force loading testing, the voltage changes in response to the applied forces at the sensing element −X become more pronounced with a sensitivity of 1.2 × 10^−3^ Volt/N due to the force application direction, as shown in Figure 8c. The sensitivity of the sensing element at the +X axis slightly increased to 2 × 10^−5^ Volt/N, and the +Y axis’s sensing element’s sensitivity remained fixed compared to the 45° test. An average sensitivity of the two former sensing elements was approximately observed at the −Y axis sensing element.

The static loading experiments were conducted without preload forces, which resulted in a negative voltage reading at low forces due to the loss of complete contact between the 3D flexible sensor’s host silicon matrix and sensing elements. The average sensitivities for the four sensing elements at the three loading forces with angles of zero degrees, 45°, and 90° were 4.2 × 10^−4^, 1.77 × 10^−4^, and 4.5 × 10^−4^ Volt/N, respectively. The sensing elements showed satisfactory sensitivity to the variation in the applied force at different engagement force angles. Some sensing elements were less sensitive to different loading scenarios due to the loading conditions. It was observed that the 3D flexible sensor showed an opposite trend at the initial contact, approximately under 5 N in all loading scenarios. That was affected by the structure configuration and the fixable materials of the proposed flexible 3D force.

### 4.2. Dynamic Loading Results

**Figure 9:**
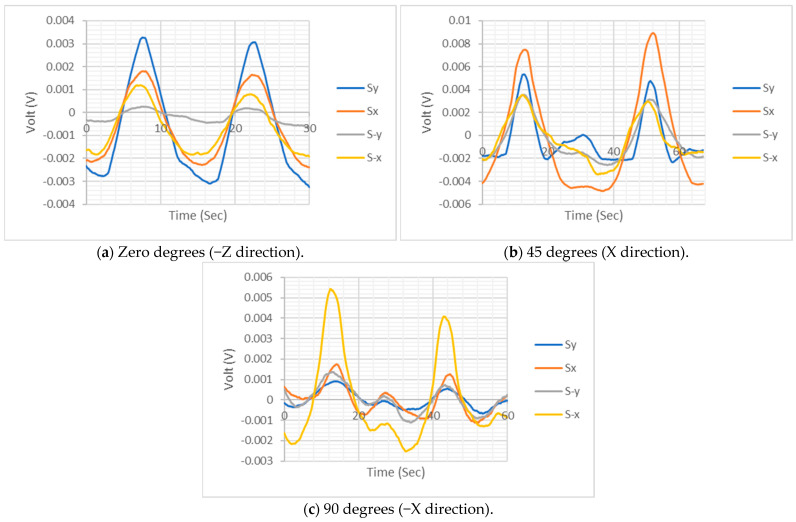
The flexible 3D force sensor dynamic loading results.

The proposed 3D flexible force sensor was tested under dynamical loading forces at 0°, 45°, and 90°, as shown in Figure 9. To ensure complete contact between the sensor and the loading head, an initial force of 5 N was applied to the sensor at the first two conditions, and approximately 3 N of contact force was applied at the shear force loading test. At each test, the averages of the four sensing elements’ voltage measurements were removed to ease the measurement analysis regardless of the constant DC offset’s effect. The rising voltage measurements identify the applied compression force, while the falling voltage readings demonstrate the compression release and tension forces. When compression force was applied to the sensor, all four sensing elements tracked the load variation during the loading test, as shown in Figure 9a. The sensing element at the −Y axis was found to be the least sensitive, while the remaining sensing elements’ sensitivity was prominent. A similar performance was observed when an inclined force was applied at 45° in the X axis direction; however, there was an intermediate voltage protuberance between the two loading cycles at the three sensing elements on the −X, Y, and −Y axes, as shown in Figure 9b. The reason for this phenomenon was the possibility of intermittent slippage during loading. This phenomenon was pronounced in the shear loading scenario in the −X axis direction for all sensing elements, as shown in Figure 9c. Nevertheless, the 3D flexible sensor sustained its effectiveness in measuring the loading cycle with good sensitivity. In addition, the sensor identified the loading directions with great sensitivity when the applied forces were not perpendicular to the sensor.

The performance of the proposed flexible 3D force sensor was investigated under different loading conditions. The sensor was sensitive to static and dynamic forces at different engagement angles. Therefore, the sensor can be used in different real-world industrial and medical applications where flexibility and 3D force measurements are required. This sensor has excellent potential to be implemented in a soft inspection robot where external forces are needed for motion control or obstacle avoidance. For medical applications, the sensor can be utilized as an artificial skin for physical therapists during training to assess their deliverable forces to patients. The noise was present at static and dynamic measurements that might originate from the dominant tunneling resistance, which is susceptible to temperature noise [5]. In addition, weak adhesion between the sensing element and the silicon rubber might introduce noise to the strain signals. Future work should focus on driving the calibration matrix, which relates the applied forces to the applied strains on the flexible 3D force sensor. In addition, the proposed sensor performance can be compared with standard 3D force sensors and drive the relationship between the input and output (transfer function). The proposed sensor characteristics, including accuracy, precession, and force range, can be investigated using the proper calibration matrix. In this study, the focus was to introduce a novel flexible 3D force sensor and investigate the sensor sensitivity under multidirectional forces.

## 5. Conclusions

In this work, a flexible 3D force sensor was developed based on a polymer nanocomposite sensing element. The PVDF/MWCANT was selected, and the sensing element film was fabricated using a spray coating. The fabricated sensing elements utilized flexible electrodes and enameled copper wire to enhance the sensor flexibility under strains. Silicone rubber was used as the primary structural material of the proposed sensor, where four sensing elements were sandwiched between two layers of silicone rubber. The sensing element distribution utilized the conventional four-beam 3D force sensor’s sensing placements. The proposed sensor was tested using static loading, and the sensing elements demonstrated great force sensitivity at the three different force engagement angles. The flexible 3D force sensor demonstrated comparable average sensitivity of 4.2 × 10^−4^ and 4.5 × 10^−4^ Volt/N under normal and shear loading static loading testing, respectively. The −X sensing element was the most sensitive, with a sensitivity of 1.2 × 10^−3^ Volt/N under shear force in the −X axis direction. That is due to the accusive shear force applied to the former sensing element. Meanwhile, the sensing element on the X axis recorded the least sensitivity of 2 × 10^−5^ Volt/N among other sensing elements in the −X shear force testing. In addition, dynamical loads with the same angles were applied to the sensor to investigate the sensor’s performance for practical applications. The sensor was able to track the loading cycle with high sensitivity, with the ability to distinguish the load direction with higher voltage change under the shear loading scenario. These results demonstrate the sensor’s effectiveness in different applications, including robotics, medical, and human–machine interface applications. In addition, the dynamic sensor’s response proves that the sensor can be employed in other applications such as impact tests, vibration monitoring, physical therapy treatment tracking, and sports performance tracking equipment.

## Data Availability

The raw data supporting the conclusions of this article will be made available by the authors on request.
